# Incidence of Clinical Outcomes in Minimally Invasive Valvular Surgery at the Ignacio Chávez National Institute of Cardiology

**DOI:** 10.7759/cureus.69859

**Published:** 2024-09-21

**Authors:** Erik F Gardner-Hilbert, Mario Gómez-Sánchez, Mario I Lumbreras-Márquez, Iñigo Díaz-Moreno

**Affiliations:** 1 Research, Panamerican University, Mexico City, MEX; 2 Cardiothoracic Surgery, Instituto Nacional de Ciencias Médicas y Nutrición Salvador Zubirán, Mexico City, MEX; 3 Epidemiology and Public Health, Panamerican University, Mexico City, MEX

**Keywords:** adult cardiac surgery, cardiothoracic surgery, heart valve disease, heart valve replacement, minimally invasive surgery

## Abstract

Minimally invasive cardiac valve replacement surgery (MICS) is a technique that has reported equivalent rates of mortality and reintervention when compared to conventional median sternotomy (CS). Additionally, MICS has inconsistently been reported to be associated with fewer postoperative complications, better cosmetic outcomes, and shorter hospital stays at the expense of longer surgical time, aortic clamp time, and extracorporeal circulation time. When comparing populations undergoing MICS vs CS at the Ignacio Chávez National Institute of Cardiology (INCICh), it was proven that there is a longer surgical, extracorporeal circulation, and aortic clamp durations in the MICS intervention, but no statistically significant difference in global mortality. MICS was also associated with a shorter hospital stay and less surgical discomfort. MICS can be considered an alternative and equivalent approach to CS for patients undergoing aortic and mitral valve replacement surgery in the Mexican population.

## Introduction

Heart valve disease is an important cause of morbidity and mortality and is a leading cause of heart failure [1]. Its incidence increases with age, and according to the Centers for Disease Control and Prevention (CDC), it affects 2.5% of the US population, and cases are expected to increase with greater global life expectancy. The most frequent valvular disease worldwide is aortic stenosis with calcific aortic valve stenotic disease (AVSD) being its most frequent presentation [2]. Addressing heart valve disease is imperative to prevent the worsening of heart function and cardiac insufficiency.

For severe valve disease, the treatment of choice is surgery [3,4], where the valve or valves may be replaced or repaired. Traditionally, this procedure is done through a total median sternotomy or conventional sternotomy (CS) [5,6]. In recent years, alternatives to the median sternotomy have been developed, such as minimally invasive cardiac surgery (MICS) techniques which require smaller incisions and less tissue manipulation and have consistently demonstrated equivalent rates of mortality and re-interventions when compared to total median sternotomies. Such small incisions are usually mini thoracotomies, “J” or “L” hemi sternotomies, and robotic surgery. In addition, it has been consistently reported that minimally invasive interventions are associated with lower bleeding rates, postoperative pain, infectious and pulmonary complications, hospitalization time, and better cosmetic results. These positive results come at the expense of longer surgical procedures, aortic clamping time, and extracorporeal circulation time [7-9].

Eight years after the implementation of these novel techniques at the National Institute of Cardiology (INCICh), there is no data regarding the outcomes of patients in the Mexican population. We believe it is justifiable to prove that these techniques are not inferior to CS which would open the door to its use nationwide. By proving its safety and positive results, more surgeons would be compelled to become proficient in these advanced procedures and it would justify the need for including these skills in cardiothoracic surgery residency programs in Mexico. Little information is available about minimally invasive surgery in Mexico which creates uncertainty and doubt among surgeons regarding safety and efficacy.

## Materials and methods

Through an observational retrospective study of cohorts, we sought to demonstrate that minimally invasive heart valve surgery had a lower incidence of mortality in patients with aortic and mitral valve disease who were operated on at the INCICh. A comparison was made between patients who underwent conventional sternotomy during the same time frame and both groups were randomly selected through convenience sampling. Previous authorization by the research committee of the INCICh was granted in order to proceed with the analysis of patients operated on at the INCICh within the timeframe of January 2015 and May 2022.

The inclusion criteria were patients ≥18 years of age who had undergone aortic valve replacement (AVR) or mitral valve replacement (MVR), by a minimally invasive technique (MICS) or by conventional median sternotomy (CS), at the INCICh from January 2015 to May 2022. Those who underwent additional coronary revascularization and surgical repair and those with disease involving ≥2 valves were excluded. One-hundred and thirty-four patients were analyzed: 67 (50%) in the MICS group (54 (80.6%) aortic valve surgery; 13 (19.4%) mitral valve surgery) and 67 (50%) in the CS group (47 (70.1%) aortic valve surgery; 20 (29.9%) mitral valve surgery).

Mortality was studied as the primary outcome, consisting of mortality in the intraoperative period, early postoperative period (<30 days), and late postoperative period (>30 days). As secondary outcomes, the total postsurgical hospitalization time was evaluated, defined as the time elapsed from the surgical intervention to hospital discharge due to improvement, the reduction in perioperative bleeding, and the total surgical time elapsed. Statistical analysis was performed using STATA version 15.1 (StataCorp. 2017. Stata Statistical Software: Release 15. College Station, TX: StataCorp LLC). 

As a primary objective, we sought to measure the overall mortality in both groups, which would be the sum of the surgical mortality, mortality in the first 30 days (early mortality) and mortality after 30 days (late mortality). As secondary objectives, we compared the differences in surgical bleeding, total surgical time and hospitalization time between the two modalities of surgery.

## Results

Table 1 shows the baseline characteristics of both groups with the means for each one, as well as the standardized difference between the two groups for each variable. Cohen described that standardized differences in the ranges of 0.2, 0.5, and 0.8 represent small, medium, and large differences respectively between two groups [10]. Since none of our results showed a result >0.5, it is safe to say that our two groups are very similar.

**Table 1:** Baseline characteristics between both groups. SD represents standard deviation, NYHA represents New York Heart Association, ACS represents acute coronary syndrome, a represents a missing value, MICS represents minimally invasive cardiac surgery, and CS represents conventional sternotomy.

	MICS	CS	Standardized differences
Variable	N=67	N=67	
Women, n (%)	27 (40.3)	31 (46.3)	0.12
Age, mean (SD)	56.93 ± 12.72	57.34 ± 11.90	0.033
NYHA, n (%)			0.166
I	16 (23.9)	15 (22.4)	
II	33 (49.3)	38 (56.7)	
III	15 (22.4)	12 (17.9)	
IV	3 (4.5)	2 (3.0)	
EuroSCORE II. mean (SD)a	1.47 ± 0.98	1.94 ± 2.24	0.271
Type 2 diabetes, n (%)	12 (17.9)	19 (28.4)	0.249
Chronic hypertension, n (%)	27 (40.3)	27 (40.3)	0
Chronic kidney disease, n (%)	1 (1.5)	1 (1.5)	0
Coronary disease, n (%)	7 (10.4)	3 (4.5)	0.288
Previous ACS, n (%)	3 (4.5)	0 (0.0)	0.306
Arrhythmias	9 (13.4)	12 (17.9)	0.123
Chronic pulmonary hypertension, n (%)	5 (7.5)	3 (4.5)	0.126
Pulmonary disease, n (%)	3 (4.5)	5 (7.5)	0.126
Type of procedure, n (%)			0.17
Mitral	15 (22.4)	20 (29.9)	
Aorta	52 (77.6)	47 (70.1)	
Emergency procedure. N (%)	5 (7.5)	4 (6.0)	0.059

Table 2 shows primary and secondary results for both groups, including conversions which translates to an intention-to-treat analysis. Table 2 is divided into two sections, the first one being the result of the primary objective of the study and the second showing the results of the secondary objectives that were established. 

**Table 2:** Primary and secondary results. CI represents confidence interval, a represents a missing value, b represents two missing values, c represents four missing values, d represents 41 missing values, e represents nine missing values, f represents packed red blood cells, cryoprecipitate, fresh frozen plasma, g represents relative risk, h represents geometric mean ratios, i represents adjusted relative risk (adjusted for age, elective vs emergency procedure, EuroSCORE II, NYHA, type 2 diabetes mellitus, chronic hypertension, chronic kidney disease and previous acute coronary syndrome), j represents adjusted geometric mean ratio (adjusted for age, elective vs emergency procedure, EuroSCORE II, NYHA, type 2 diabetes mellitus, chronic hypertension, chronic kidney disease and previous acute coronary syndrome), MICS represents minimally invasive cardiac surgery, and CS represents conventional sternotomy.

	MICS	CS	Raw effect	Adjusted effect	p-value
Result	N=67	N=67	CI 95%	CI 95%	
Primary					
General mortality, n (%)a	6 (9.0)	4 (6.1)	1.76 (0.40, 7.70)g	1.39 (0.35, 5.50)i	0.448
Intraoperative mortality, n (%)	2 (3.0)	0 (0.0)			
<30 days mortality, n (%)	3 (4.5)	2 (3.0)	6.71 (0.38, 117.40)g	5.70 (0.33, 97.88)i	
>30 days mortality, n (%)	1 (1.5)	2 (3.0)	2.08 (0.06, 63.39)g	1.59 (0.16, 15.67)i	
Secondary					
Early reintervention, n (%)	7 (10.4)	10 (14.9)	0.67 (0.21, 2.07)g	0.76 (0.31, 1.83)i	
Bleeding in mL, geometric mean, (CI 95%)b	364 (314, 423)	314 (268, 367)	1.16 (0.94, 1.44)h	1.14 (0.92, 1.42)j	
Total surgical time geometric mean (CI 95%)b	278 (256, 301)	244 (230, 260)	1.16 (1.05, 1.28)h	1.13 (1.02, 1.24)j	
Time on extracorporeal circulation, geometric mean (CI 95%)	129 (117, 143)	97 (91, 104)	1.36 (1.21, 1.54)h	1.31 (1.17, 1.47)j	
Aortic clamp time, geometric mean (CI 95%)	90 (81, 100)	72 (67, 77)	1.27 (1.12, 1.45)h	1.23 (1.09, 1.40)j	
Time intubated, geometric mean (CI 95%)d	932 (524, 1655)	929 (613, 1407)	1.01 (0.47, 2.17)h	1.02 (0.47, 2.24)j	
Transfused blood products, n (%)e, f	61 (96.8)	56 (90.3)	2.89 (0.52, 16.02)g	1.08 (0.97, 1.19)i	
Transfused blood products, geometric mean (CI 95%)e, f	3.52 (2.99, 4.15)	3.48 (2.93, 4.15)	1.05 (0.82, 1.36)h	1.03 (0.80, 1.33)j	
Composite results of complications, n (%)	34 (50.7)	34 (50.7)	1.06 (0.51, 2.21)g	1.02 (0.72, 1.43)i	
Respiratory, n (%)	10 (14.9)	9 (13.4)	1.37 (0.47, 3.95)g	1.21 (0.52, 2.81)i	
Infection, n (%)	10 (14.9)	12 (17.9)	0.69 (0.25, 1.88)g	0.80 (0.37, 1.75)i	
Intraoperative MI, n (%)	2 (3.0)	1 (1.5)	2.12 (0.17, 25.78)g	1.98 (0.19, 20.67)i	
Intraoperative stroke, n (%)	0 (0.0)	2 (3.0)			
Deep vein thrombosis, n (%)	2 (3.0)	1 (1.5)	2.45 (0.14, 42.24)g	2.02 (0.24, 16.42)i	
Acute kidney injury, n (%)	3 (4.5)	6 (9.0)	0.36 (0.06, 2.01)g	0.40 (0.08, 1.89)i	
Arrhythmias, n (%)	20 (29.9)	18 (26.9)	1.31 (0.58, 2.93)g	1.18 (0.70, 1.98)i	
Days hospitalized, geometric mean (CI 95%)	9.5 (7.97, 11.33)	11.85 (10.30, 13.64)	0.80 (0.62, 1.01)g	0.82 (0.64, 1.04)j	

During the analysis of the results in Table 2, a logistic regression was performed for categorical variables and a linear regression for continuous variables to adjust the results to the confounding factors described in the literature [11,12]. In this study, our results were adjusted for EuroSCORE II, NYHA classification, type 2 diabetes (T2DM), chronic hypertension (HTN), chronic kidney disease (CKD), age and emergency vs elective procedure. Dichotomous variables are reported as relative risk (RR) and continuous variables as geometric mean ratios. The p-value was only calculated for the general mortality since this is our primary hypothesis and result. If we were to calculate p-values for all variables, we would have a higher chance of obtaining a false positive result. The general mortality was not statistically significant (p=0.638); however, we did observe prolonged surgical (13%), extracorporeal circulation (31%), and aortic clamp durations (23%) in the MICS group. The percentages were obtained from the adjusted effect of each result and represent how much longer the times were in the MICS vs the CS group.

Table 3 shows the primary and secondary results excluding the surgical conversions between groups. The analysis in this sense was per protocol. The results were similar to those shown in Table 2, showing longer surgical (11%), extracorporeal circulation (28%), and aortic clamp durations (21%). The general mortality result was also not statistically significant (p=0.718).

**Table 3:** Primary and secondary results excluding conversions. CI represents confidence interval, a represents a missing value, b represents two missing values, c represents four missing values, d represents 41 missing values, e represents nine missing values, f represents packed red blood cells, cryoprecipitate, fresh frozen plasma, g represents relative risk, h represents geometric mean ratios, i represents adjusted relative risk (adjusted for age, elective vs emergency procedure, EuroSCORE II, NYHA, type 2 diabetes mellitus, chronic hypertension, chronic kidney disease and previous acute coronary syndrome), j represents adjusted geometric mean ratio (adjusted for age, elective vs emergency procedure, EuroSCORE II, NYHA, type 2 diabetes mellitus, chronic hypertension, chronic kidney disease and previous acute coronary syndrome), MICS represents minimally invasive cardiac surgery, and CS represents conventional sternotomy.

	MICS	CS	Raw effect	Adjusted effect	p-value
Result	N=63	N=67	CI 95%	CI 95%	
Primary					
General mortality, n (%)a	3 (4.8)	4 (6.1)	0.78 (0.18, 3.39)g	0.77 (0.19, 3.08)i	0.718
Intraoperative mortality, n (%)	1 (1.6)	0 (0.0)	-	-	
<30 days mortality, n (%)	2 (3.2)	2 (3.0)	1.06 (0.15, 7.37)g	2.89 (0.28, 29.25)i	
>30 days mortality, n (%)	0 (0.0)	2 (3.0)	-	-	
Secondary					
Early reintervention, n (%)	7 (10.4)	10 (14.9)	0.74 (0.30, 1.84)g	0.78 (0.32, 1.89)i	
Bleeding in mL, geometric mean, (CI 95%)b	349 (301, 406)	314 (268, 367)	1.11 (0.89, 1.38)h	1.12 (0.90, 1.39)i	
Total surgical time geometric mean (CI 95%)b	267 (250, 286)	244 (230, 260)	1.09 (0.99, 1.19)	1.11 (1.01, 1.21)j	
Time on extracorporeal circulation, geometric mean (CI 95%)	123 (113, 134)	97 (91, 104)	1.26 (1.14, 1.40)h	1.28 (1.15, 1.42)j	
Aortic clamp time, geometric mean (CI 95%)	87 (79, 96)	72 (67, 77)	1.20 (1.06, 1.35)h	1.21 (1.06, 1.37)j	
Time intubated, geometric mean (CI 95%)d	787 (466, 1329)	929 (613, 1407)	0.84 (0.44, 1.62)h	0.79 (0.40, 1.58)i	
Transfused blood products, n (%)e, f	57 (96.6)	56 (90.3)	1.06 (0.97, 1.17)g	1.07 (0.97, 1.19)i	
Transfused blood products, geometric mean (CI 95%)e, f	3.53 (2.99, 4.17)	3.48 (2.93, 4.15)	1.01 (0.79, 1.28)h	1.05 (0.81, 1.36)i	
Composite results of complications, n (%)	31 (49.2)	34 (50.7)	0.96 (0.68, 1.37)g	0.96 (0.68, 1.37)i	
Respiratory, n (%)	8 (12.7)	9 (13.4)	0.94 (0.38, 2.30)g	1.02 (0.41, 2.56)i	
Infection, n (%)	8 (12.7)	12 (17.9)	0.70 (0.30, 1.62)g	0.66 (0.28, 1.54)i	
Intraoperative MI, n (%)	1 (1.6)	1 (1.5)	1.06 (0.06, 16.82)g	-	
Intraoperative stroke, n (%)	0 (0.0)	2 (3.0)	-	-	
Deep vein thrombosis, n (%)	2 (3.2)	1 (1.5)	2.12 (0.19, 23.09)g	2.02 (0.24, 16.74)i	
Acute kidney injury, n (%)	3 (4.8)	6 (9.0)	0.53 (0.13, 2.04)g	0.42 (0.09, 1.96)i	
Arrhythmias, n (%)	17 (27.0)	18 (26.9)	1.00 (0.56, 1.77)g	1.05 (0.60, 1.82)i	
Days hospitalized, geometric mean (CI 95%)	9.74 (8.47, 11.20)	11.85 (10.30, 13.64)	0.82 (0.67, 1.00)h	0.81 (0.65, 1.01)i	

Table 4 shows the analysis of the primary and secondary results including conversions. Nonparametric tests were used to obtain these results. For dichotomous variables, Fisher's test was used and for continuous variables, the Wilcoxon-Mann-Whitney test was used. The data was expressed as a mean and an interquartile range (IQR). A sensitivity analysis can be performed given that the type of test used does not change the results. This leads us to conclude that the data reported is robust and consistent. Longer surgical (p=0.014), extracorporeal circulation (p<0.001), and aortic clamp times (p<0.001) were observed in the MICS group during this last analysis.

**Table 4:** Primary and secondary results analyzed with nonparametric tests and including conversions. IQR represents the interquartile range, a represents a missing value, b represents two missing values, c represents four missing values, d represents 41 missing values, e represents nine missing values, f represents packed red blood cells, cryoprecipitate and fresh frozen plasma, g represents Fisher’s exact test, h represents the Wilcoxon-Mann-Whitney U-test, MICS represents minimally invasive cardiac surgery, and CS represents conventional sternotomy.

	MICS	CS	p-value
Result	N=67	N=67	
Primary			
General mortality, n (%) a, g	6 (9.0)	4 (6.1)	0.744
Intraoperative mortality, n (%) g	2 (3.0)	0 (0.0)	0.496
<30 days mortality, n (%) g	3 (4.5)	2 (3.0)	0.999
>30 days mortality, n (%) g	1 (1.5)	2 (3.0)	0.999
Secondary			
Early reintervention, n (%) g	7 (10.4)	10 (14.9)	0.605
Bleeding in mL, median (IQR) b, h	350 (220, 500)	320 (220, 480)	0.450
Total surgical time median (IQR) c, h	254 (225, 314.5)	240 (210, 276)	0.014
Time on extracorporeal circulation, median (IQR) h	118 (101, 157)	94 (83, 120)	<0.001
Aortic clamp time, median (IQR) h	91 (75, 110)	71 (59, 91)	<0.001
Time intubated, median (IQR) d, h	360 (0, 750)	435 (0, 1440)	0.280
Transfused blood products, n (%) e, f, g	61 (96.8)	56 (90.3)	0.164
Transfused blood products, median (IQR) e, f, h	4 (3, 5)	3 (2, 5)	0.400
Composite results of complications, n (%) g	34 (50.7)	34 (50.7)	0.999
Respiratory, n (%) g	10 (14.9)	9 (13.4)	0.999
Infection, n (%) g	10 (14.9)	12 (17.9)	0.816
Intraoperative MI, n (%) g	2 (3.0)	1 (1.5)	0.999
Intraoperative stroke, n (%) g	0 (0.0)	2 (3.0)	0.496
Deep vein thrombosis, n (%) g	2 (3.0)	1 (1.5)	0.999
Acute kidney injury, n (%) g	3 (4.5)	6 (9.0)	0.492
Arrhythmias, n (%) g	20 (29.9)	18 (26.9)	0.848
Days hospitalized, median (IQR) h	9 (7, 13)	11 (8, 14)	0.230

## Discussion

As shown by Kuinose et al. in a previous study, we found longer surgical, extracorporeal circulation, and aortic clamping durations for MICS, as well as a decrease in total days hospitalized and less postoperative discomfort [13]. After reviewing and analyzing the data, we found that the difference was not statistically significant.

Regarding surgical duration, as expected, the MICS approach is more complex, offers fewer viewing angles, and is therefore more time-consuming which translates into longer operative times. Regarding surgical duration, multiple studies have shown that after the learning curve is overcome, surgical times are greatly reduced in the MICS [14]. Although in this study we did not analyze different minimally invasive surgical techniques, surgical times also seem to differ between techniques. An example of this is seen in a meta-analysis done by Phan et al., which showed that a minithoracotomy approach was associated with longer surgical duration due to greater technical difficulty while maneuvering long-shaft instruments [15]. Regarding postsurgical complications, we found a higher incidence of atelectasis, pneumonia, myocardial infarction, and vascular complications in the MICS group, although none of them were statistically significant.

Intubation duration analysis was excluded from our study due to insufficient data. This is a parameter that is important to compare between traditional and minimally invasive techniques since this directly prolongs the length of stay in an intensive care unit and the total days spent in a hospital. In our literature review, we did not find statistically significant data suggesting that MICS is associated with less intubation and artificial ventilatory requirements [8,9,15,16].

Although our data regarding hospital length of stay was not statistically significant, there was a strong trend toward fewer days spent at the hospital for patients who underwent MICS. This trend is consistent with previous meta-analyses and systematic reviews that report fewer hospitalization days as well as days spent in the ICU [17,18].

In the intention-to-treat analysis, which includes conversions to CS, we observed a difference in general mortality in the MICS group which was not statistically significant. This may be due to the longer surgical times which increases the risk of complications like thrombosis, stroke, infections, etc. Multiple studies have shown the same complications when comparing MICS to CS and these were attributed to longer times spent on ECMO, aortic clamp duration, and even to the surgical technique e.g. transseptal approach to reach the mitral valve [9,19]. The vascular complications could be due to the peripheral (femoral) cannulation method used in these techniques for extracorporeal circulation, and myocardial infarctions appear to be unrelated to the technique. In general, there is a nonsignificant difference in mortality, this being greater for the minimally invasive group. We believe that this may be also due to a longer learning curve since MICS requires more experience and years of training, which translates into a more prolonged surgical duration.

As Di Bacco et al. explained, minimally invasive surgery could be of great value for patients who suffer from obesity, COPD, or patients who as described as being frail. MICS reduces the amount of trauma that the body must sustain during an already metabolically demanding procedure by reducing incision size, movement, and manipulation of tissue and time spent in the operating room [20]. A considerable percentage of our patient population presented some kind of disease that would increase their risk of perioperative complications, which is why undergoing minimal manipulation and stress benefits this patient population.

One limitation that was sought to be overcome was the small sample size that skewed many of the results; it was therefore proposed to include more patients in this protocol and thus increase the power of the results. Even with this study’s small population, our results regarding surgical, extracorporeal circulation, and aortic clamp durations were consistent with different types of analysis found in other publications and this creates great certainty and consistency in our results. Despite this, we believe that our study could be enriched by becoming a multicenter study and a long-term follow-up would also be of great value to determine the incidence of re-interventions, recovery time, and cosmetic satisfaction from the patients. 

## Conclusions

At the INCICh, we found no statistically significant difference in the incidence of mortality between MICS and CS during the study period. It was determined that although both surgical techniques are equally safe at the INCICh, total surgery, extracorporeal circulation, and total aortic clamping times were longer in the MICS group than in the CS group.

This study could benefit from a larger sample size to reach more robust results and conclusions about the benefits of minimally invasive surgery. This study was able to prove that at the INCICh, the outcomes for both groups were very similar to those described in the literature and could benefit from a multicenter analysis to compare the results of the INCICh to those of other institutions.
